# Selection of Tumor-Specific Cytotoxic T Lymphocytes in Acute Myeloid Leukemia Patients Through the Identification of T-Cells Capable to Establish Stable Interactions With the Leukemic Cells: “Doublet Technology”

**DOI:** 10.3389/fimmu.2018.01971

**Published:** 2018-09-03

**Authors:** Estefanía García-Guerrero, Luís I. Sánchez-Abarca, Esther Domingo, Teresa L. Ramos, Jose A. Bejarano-García, Jose A. Gonzalez-Campos, Teresa Caballero-Velázquez, Jose A. Pérez-Simón

**Affiliations:** ^1^Instituto de Biomedicina de Sevilla, UGC de Hematología, Hospital Universitario Virgen del Rocío and Consejo Superior de Investigaciones Científicas (CSIC) and Centro de Investigación Biomédica en Red Cáncer (CIBERONC), Universidad de Sevilla, Seville, Spain; ^2^Servicio de Hematología, Instituto de Investigación Biomédica de Salamanca (IBSAL) – Hospital Universitario de Salamanca, Salamanca, Spain

**Keywords:** immunotherapy, tumor-specific T cells, acute myeloid leukemia, cell selection, T cell-tumor cell synapse

## Abstract

The relevance of the immune system in cancer has long been studied. Autologous adoptive T cell therapies, based on the use of tumor infiltrating lymphocytes (TILs), have made great progress in recent years for the treatment of solid tumors, especially melanoma. However, further work is needed to isolate tumor-reactive T cells among patients diagnosed with hematologic malignancies. The dynamics of the interaction between T cells and antigen presenting cells (APC) dictate the quality of the immune responses. While stable joints between target cells and T lymphocytes lead to the induction of T cell activation and immune response, brief contacts contribute to the induction of immune-tolerance. Taking advantage of the strong interaction between target cell and activated T-cells, we show the feasibility to identify and isolate tumor-specific cytotoxic T lymphocytes (CTLs) from acute myeloid leukemia (AML) patients by flow cytometry. Using this technology, CTLs bound through T cell receptor (TCR) to tumor cells can be identified in peripheral blood and bone marrow and subsequently selected and isolated by FACS-based cell sorting. These CTLs display higher percentage of effector cells and marked cytotoxic activity against AML blasts. In conclusion, we have developed a new procedure to identify and select specific cytotoxic T cells in patients diagnosed with acute myeloid leukemia.

## Introduction

Adoptive T cell therapy (ACT) is a potentially powerful immunotherapeutic approach to cancer treatment that relies on the infusion of tumor-specific cytotoxic T lymphocytes (CTLs) into the patient (1–3). Cancer immunotherapy with tumor-reactive T cells has shown remarkable responses in patients (3–7). In fact, it was convincingly shown that tumor-infiltrating lymphocytes (TILs) selected for their reactivity toward autologous melanoma cells displayed high functional activity yielding 50% objective responses in metastatic melanoma patients (8–11). TIL therapy is now explored in other cancers than melanoma (12–15), demonstrating that this approach is both feasible and efficacious in solid tumors; however, further work is needed to isolate tumor-reactive T cells in hematologic malignancies, especially in acute myeloid leukemia (AML).

To date, two approaches are widely applied for the detection and isolation of tumor-specific cytotoxic T lymphocytes: The first is based on the assessment of specific T cells functions, such as cytokine (typically IFN-γ) production (16) or activation-induced phenotypical modifications, such as cell surface expression of CD107a (LAMP-1) and / or CD137 (4-1BB) (17). IFN-γ production is the most commonly used variable to detect T-cell reactivity against antigen-presenting targets. However, cytokine-producing CD8+ T cells are not exclusively cytotoxic (18, 19), and consequently in the therapeutic context, it is important to distinguish T-cell reactivity from functional cytotoxicity, specifically the capacity of CTLs to destroy target cells. An alternative strategy uses soluble peptide-major histocompatibility complex (pMHC) multimers consisting of multiple pMHC complexes that have been chemically linked together and conjugated to a detectable marker to identify and separate antigen-specific T cells from the whole lymphocyte population (20–22). However, this technology has several obstacles that need to be solved. For example, the binding affinity threshold for pMHC class I (pMHC-I) tetramers is significantly higher than that required for T cell activation. As a result, pMHC-I tetramers cannot be used to detect all antigen-specific CD8+ T cells (22, 23). In addition, the use of MHC class II-based reagents to obtain antigen-specific CD4+ T cells is still challenging due to the lower affinity of pMHC-II tetramers-TCR interactions (22, 24). Thus, there is currently a pressing need to extend pMHC multimer technology to a point where it can be used to stain all antigen-specific T cells in any biological systems. Although both approaches have been successfully used to obtain antigen-reactive T cells, the need for *a priori* knowledge of the exact tumor antigen is a major limiting factor, since the target antigen is not well known for most tumor cells. To address this technology gap, we have developed a new method for obtaining tumor-specific CTLs without the need of *a priori* knowing the exact tumor antigen.

The specificity of T cell activation depends on the interaction of peptide-MHC complexes and TCR (25). A kinetic model has been proposed that states that T cell signaling is highly dependent on the dissociation rate of pMHC from TCR. In this model, pMHC-TCR complexes with slow dissociation rates send positive signals to T cell, whereas fast off-rates result in negative signaling (26–29). This model explains the experimentally observed relationship between T cell function and dissociation rate of ligand from receptor in numerous reports (30). In this sense, we have published that the pHLA-TCR interactions that involve immune reactive peptides are more stable and strong than those which do not induce a triggering of the TCR activation. In our model, the stabilization of the reactive complex was achieved due to less fluctuations and more salt bridges comparing with non-reactive peptides (31).

In this report, we develop a new procedure to identify and isolate functional tumor-specific cytotoxic T lymphocytes from acute myeloid leukemia (AML) patients through FACS-based cell sorting. Our results show that tumor-reactive T cells from AML patients can be identified and isolated based on their capability to form stable and strong interactions through TCR with tumor cells (doublet-forming T cells), as well as they show cytolytic activity against primary AML cells.

## Materials and methods

### Human samples

Buffy coats were kindly donated by the Regional Centre for Blood Transfusions at Virgen del Rocío University Hospital, Seville (Spain). Peripheral blood and bone marrow samples were obtained from AML patients. The local ethics committee provided institutional review board approval for this study, and informed consent was obtained from all donors in accordance with the Declaration of Helsinki.

### PBMC purification

Peripheral blood mononuclear cells (PBMC) were isolated from healthy donors or AML patients in complete remission status (CR) by density gradient centrifugation using Ficoll–Paque solution (Amersham Biosciences, Uppsala, Sweden). Blood was mixed with room temperature DPBS at 8:1 ratio. Density centrifugation was performed for 30 min at 400 × g with acceleration and deceleration settings of 9 and 2, respectively. The PBMC were cultivated in a 48 well plate a final concentration of 1 × 10^6^ cells/ml.

### CD3 depletion and irradiation

CD3+ cells (T cells) were depleted from the PBMC fraction twice using CD3 MicroBeads, human (Miltenyi Biotec) following the manufacturer's instructions. Then, the CD3 depleted-PBMC (CD3-PBMC) were irradiated at 25Gy. After irradiation, CD3-PBMC were stained with a nonspecific labeling PKH-67 (PKH67GL Sigma-Aldrich) (CD3-PKH+PBMC).

### Primary co-culture

Co-cultures between PBMC from a healthy donor with target cells from a second healthy donor were performed at 3:1 ratio in a 48 well plate. The co-culture was incubated at 37°C without shaking. The cell cultures were observed under confocal microscope (Olympus BX-61) and analyzed at different time points (2, 15, 24, 48 h) by flow cytometry to study the percentage of doublet T cells (T cell bound to a target cell). The following panel was used: PKH (read in FITC channel)/ APC anti-human CD3/ V450 anti-human CD45 (BD Biosciences).

Once the best timing and culture conditions were stablished, co-cultures between PBMC from AML patients in CR and AML tumor cells from the same patient were performed to study the percentage of doublet-forming T cells in patient's samples (T cell bound to a tumor cell). Alternatively, bone marrow samples obtained from AML patients at diagnosis were maintained in a 48 well plate for 15–20 h and stained as specified below. For blocking experiments, HLA A,B,C (25 μg/ml) and αβTCR (100 μg/ml) antibodies (Biolegend) were added to the PBMCs and incubated 1 h at 37°C before the co-culture. Blocking antibodies remained in the co-culture for the duration of the experiment.

### FACS-based cell sorting

After 15 h of co-culture, cells were stained with the following antibodies: PE anti-human CD25/ APC anti-human CD3/ V450 anti-human CD45 (BD Biosciences). FACS Aria Fusion Cell Sorter was used to sorter the different populations. The sorting strategy was: First, the viable region FSC/SSC, doublet zone FSC-A/FSC-H and positive region for CD45 was selected. Then, double positive cells (CD3+PKH+) and non-doublet cells (CD3+PKH-) were gated. Finally, within the non-doublet cells (CD3+PKH-), two different populations were sorted using the antibody CD25. The nozzle size used to sorter the doublet cells was 85 μm.

### Immunophenotype

Doublet/non-doublet T cells were analyzed by flow cytometry and groups were compared regarding CD4+/CD8+ proportions, naïve/effector/central memory/effector memory proportions, cytotoxic markers and regulatory markers. Flow analyses were performed on a BD FACSCanto II and data analyzed using Infinicyt v1.7 software (Cytognos, Salamanca, Spain).

### Secondary co-cultures

#### Cytotoxicity assay

Secondary co-cultures were performed in a 96 well plate at 37°C. Tumor cells from the same patient (used in primary co-culture) were stained with PKH-67 or a tumor marker. Co-cultures of patient's doublet /non-doublet T cells with autologous tumor cells were maintained for 7 h. The cytotoxic activity of doublet population vs. non-doublet population was analyzed by flow cytometry using Annexin V / 7AAD staining. The specific lysis was calculated following the next formula: [(target viability alone–target viability with doublet or non-doublet T cells)/ target viability alone] x100. The viability ratio was calculated as follows: 1-[(tumor viability alone–tumor viability with doublet or non-doublet cells)/ tumor viability alone].

#### Suppression assay

To test regulatory function, co-cultures of non-doublet T cells with activated conventional T cells were performed. Conventional T cells (CD3+ cells) were purified by positive isolation using Miltenyi MACS MicroBeads and magnetic cell separation protocol according to manufacturer's instruction. The conventional T cells were stained with PKH-67 and stimulated with plate bound anti-CD3 (10 μg/mL) and soluble anti-CD28 (1 μg/mL) mAbs (BD Biosciences). An increasing proportion of conventional T cells was used for studying the inhibition function of non-doublet T cells. After 4 days, cells were collected, stained with APC anti-human CD3/ 7AAD/ V450 anti-human CD25/ V500 anti-human CD45 mAbs (BD Biosciences) and analyzed by flow cytometry. ModFit software was used to calculate the percentage of resting and proliferating cells.

#### Activation and cytokines assays

The sorted doublet/non-doublet T cells were co-cultured with target CD3-PKH+PBMC. Moreover, the cells were activated with anti-CD3/CD28 beads (Human T-Activator, Gibco) in cell to bead ratio of 1:3 as a control and CD69 activation marker was studied by flow cytometry at 24 h after the secondary co-culture. Furthermore, INF-γ and IL-2 production was analyzed by ELISA following manufacturer's instructions (Biolegend).

### Statistical analysis

All data are presented as mean ± standard deviation/range of either absolute values or percentage. Statistical analyses were performed using Prism software v6.07 (GraphPad, San Diego, California). Statistical significance was assessed by paired Student's *t*-test. In all the tests, *p*-values were ^*^p < 0.05, ^**^p < 0.01, ^***^p < 0.001, and ^****^p < 0.0001.

## Results

### Identification and isolation of antigen-specific T lymphocytes (doublet-forming T cells) by FACS-based cell sorting

**Figure 1:**
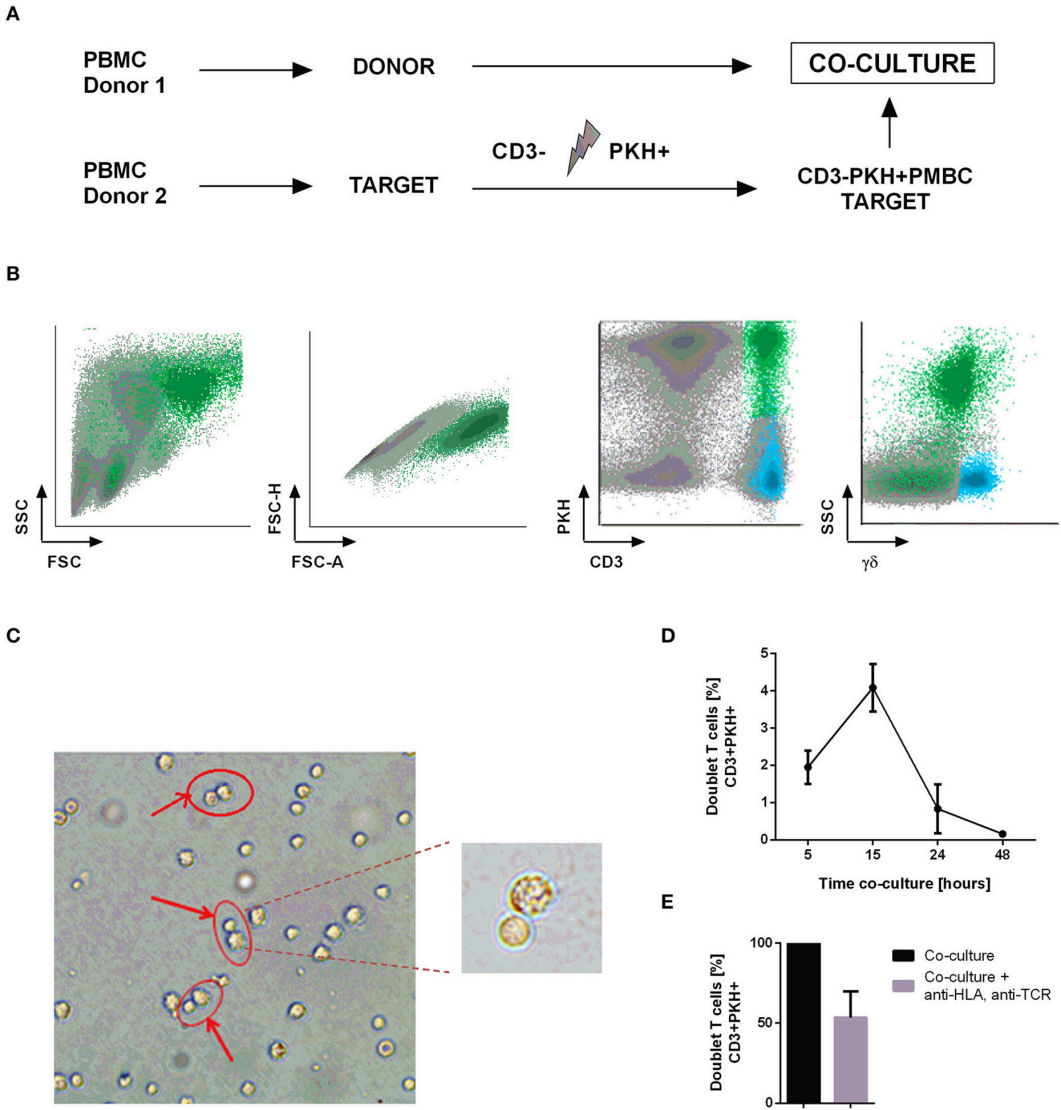
Identification and isolation of doublet-forming T cells. **(A)** Schedule of the procedure: PBMC from two healthy donors were obtained by density gradient centrifugation. Target PBMC were depleted of CD3 cells, irradiated and stained with PKH-67 (CD3–PKH+PBMC). Co-cultures of donor's PBMC and target CD3–PKH+PBMC were performed. **(B)** Doublet-forming T cells were identified by flow cytometry as a population with higher FSC/SSC, doublet zone FSC-H/FSC-A and expression of both CD3 and PKH (CD3+PKH+). **(C)** Doublet-forming T cells were also identified under confocal microscope. **(D)** Representation of the percentage of doublet T cells vs. co-culture time (hours) is shown. Depicted are the mean ± *SD* of three independent experiments. **(E)** The bar diagram shows the percentage of doublet-forming T cells (CD3+PKH+) in presence or absence of HLA-A,B,C, and αβTCR antibodies. Depicted are the mean ± *SD* of three independent experiments (performed in duplicates).

Therapies using tumor infiltrating lymphocytes (TILs) have made great progress in the treatment of solid tumors, especially melanoma (32, 33). We aimed to transfer this therapeutic approach to the treatment of hematologic malignancy. Therefore, we strived to obtain functional tumor-reactive CTLs from AML patients to subsequently use them for autologous adoptive cell transfer therapy. To achieve this goal, we began by performing co-cultures of two different healthy donors to set up the best conditions to identify and select those T cells which recognize and bind the target cell (doublet-forming T cells). In particular, co-cultures between donor 1 (Donor) and donor 2 (Target) were performed to identify doublet-forming T cells by flow cytometry indicating a T cell-target cell complex. For that purpose, target PBMC were irradiated and stained with PKH-67 to differentiate them from the donor's PBMC (Figure 1A). T-cell depletion in the target fraction allowed a better activation of donor's T cells (Figure S1A) (*n* = 4, *p* = 0.005 for CD25; *p* = 0.003 for CD69). Antigen-specific T lymphocytes (doublet-forming T cells) were identified as a population that shows a higher FSC/SSC distribution, appears in doublet zone FSC-H/FSC-A and expresses simultaneously CD3 and PKH. Thus, the doublet positive events (CD3+PKH+) by flow cytometry consist of CD3+ T cells from the donor bound to PKH-stained target cells. Interestingly, doublet T cells were negative for γδ expression suggesting that they are αβ T cells (Figure 1B). The doublet-forming T cells were also identified under confocal microscope (Figure 1C). The monitoring of co-cultures was performed using an incubator integrated with a confocal microscope and a camera. Hence, we could observe that CTLs bind to target cells (green for the PKH emission, data not shown).

After identifying the “doublet positive cells” as a population which demonstrates higher FSC/SSC distribution, appears in doublet zone FSC-H/FSC-A and positive expression of CD3 and PKH, we explored the optimal incubation time to obtain the highest proportion of doublet-forming T cells. Consequently, we performed co-cultures and analyzed the percentage of doublet population at different time points by flow cytometry. The time point of 15 h resulted in the highest doublet percentage (Figure 1D). In order to further analyze this interaction, we performed co-cultures in presence or absence of HLA A,B,C and αβTCR antibodies to block conjugate formation through TCR-HLA contact. An inhibition of doublet-forming T cells was observed in presence of blocking antibodies suggesting TCR-dependent cell interaction (Figure 1E). Once the doublet population was identified and the optimal time of co-culture determined, we continued to select this population through cell sorting. Co-cultures of donor's PBMC and CD3-depleted, PKH-stained target PBMC (CD3-PKH+PBMC) were performed. After 15 h of incubation, the cells were stained using the following panel: PE anti-human CD25/ APC anti-human CD3/ V450 anti-human CD45. Afterwards, the cells were sorted based on their FSC/SSC and FSC-A/FSC-H distribution as well as their positivity for both CD3 and PKH (doublet-forming T cells) (Figure S1B). The FACS Aria Fusion Cell Sorter was run with an 85 μm nozzle for sorting the doublet positive cells due to their large size and higher sensitivity (T cell bound to a target cell).

Doublet-forming T cells were identified in a range of 3–6% (*n* = 10). The selected populations CD3+PKH+ (doublet-forming T cells) and CD3+PKH– (non doublet-forming T cells) were further characterized. Within this latter fraction, two populations were identified: CD3+PKH–CD25– and CD3+PKH–CD25+ (Figure S1B). After the sorting procedure, cells were analyzed by flow cytometry and observed under confocal microscope. The doublet T cells (CD3+PKH+) were still forming doublet positive population (>70%, *n* = 3) and the non-doublet populations, both CD3+PKH–CD25+ and CD3+PKH–CD25–, were also highly purified (>95%, *n* = 3) (Figure S2).

### Doublet-forming T cells show higher percentage of effector cells and specific cytotoxic activity and cytokine production as compared to non-doublet T cells

**Figure 2:**
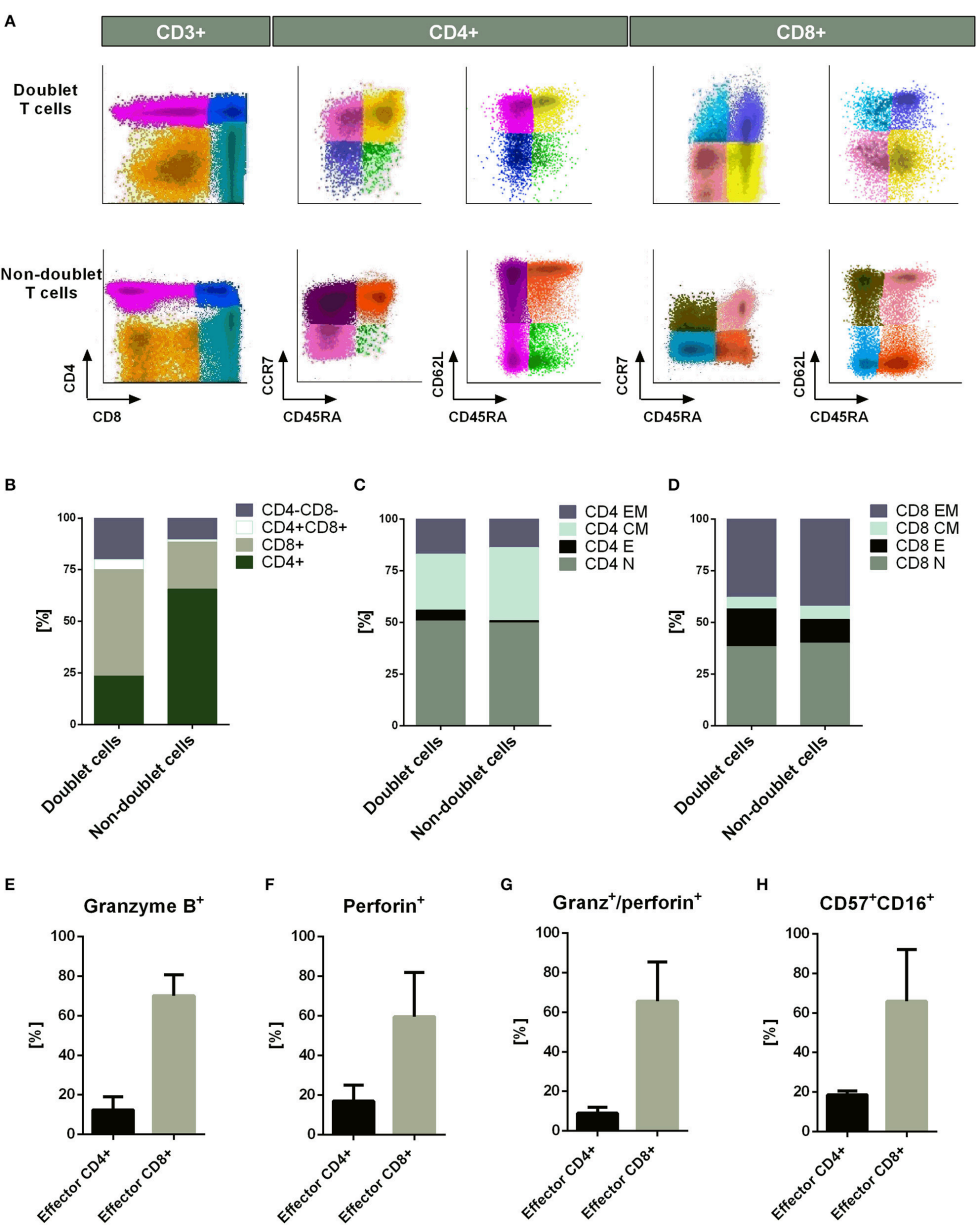
Immunophenotype of doublet-forming T cells. **(A)** Dot plots show the expression of CD4, CD8, CD45RA, CD62L, and CCR7 on doublet T cells (upper panel) and non-doublet T cells (bottom panel) from one representative case (*n* = 6 experiments). The T cell-subtypes: naïve (CD45RA+CCR7+CD62L+), effector (CD45RA+CCR7–CD62L–), central memory (CD45RA–CCR7+CD62L+) and effector memory (CD45RA–CCR7–CD62L–) were analyzed. The Infinicyt software was used for data analysis. **(B)** Percentage of CD4+, CD8+, CD4+/CD8+, and CD4–/CD8– cells in doublet T cells compared to non-doublet T cells. **(C)** Percentage of naïve, effector, central memory, and effector memory CD4+ T cells is shown. The same analysis is shown in **(D)** for CD8+ T cells. Data show mean values of six independent experiments. **(E)** The bar diagram shows the Granzyme B expression of effector CD4+ and CD8+ doublet T cells. **(F)** The bar diagram shows the Perforin expression of effector CD4+ and CD8+ doublet T cells. **(G)** The bar diagram shows the Granzyme B and perforin expression of effector CD4+ and CD8+ doublet T cells. **(H)** The bar diagram shows the CD57 and CD16 expression of effector CD4+ and CD8+ doublet T cells. **(E–H)** Data show mean values ± *SD* of three independent experiments.

Immunophenotyping analysis showed differences between doublet-forming T cells (CD3+PKH+) and those T cells which did not form stable and strong interactions with target cells (CD3+PKH–CD25–). To compare the immunophenotype of both populations, we performed co-cultures for 15 h and analyzed them by flow cytometry. We studied not only the CD4+/CD8+ ratio, but also the percentage of T cell-subtypes (Figure 2A). The T cell-subtypes were characterized by the expression of CD45RA, CD62L, and CCR7 following the next criteria: Naïve (CD45RA+CCR7+CD62L+), Effector (CD45RA+CCR7–CD62L–), Central Memory (CD45RA–CCR7+CD62L+) and Effector Memory (CD45RA–CCR7– CD62L–). Comparing doublet-forming T cells with non doublet-forming T cells, we observed differences regarding the ratio CD4+/CD8+. As shown in Figure 2B, doublet T cells displayed a higher percentage of CD8+ T cells. Within CD4+ T cells, similar percentage of naïve, central memory and effector memory between doublet cells and non-doublet cells was observed (Figure 2C). The same result was noted in CD8+ T cells (Figure 2D). However, different percentage of effector CD4+ and CD8+ T cells was observed among doublet T cells as compared to non-doublet T cells (Figures 2C,D). Accordingly, there was a significant difference in the percentage of CD4+ and CD8+ cells between doublet-forming T cells and non doublet-forming T cells (Figure S3A) (*n* = 6, *p* < 0.001). Furthermore, the percentage of effector CD4+ and CD8+ was significantly higher in doublet-forming population (Figures S3B,C) (*n* = 6, *p* < 0.001 for effector CD4+; *p* < 0.05 for effector CD8+). Next, we explored, among effector CD4+ and CD8+ cells, those with cytotoxic phenotype using Granzyme B and perforin staining as well as CD57 and CD16 markers (Figures S3D,E). As expected, a high percentage of effector CD8+ doublet T cells showed Granzyme B and perforin expression, thus corresponding with a cytotoxic immunephenotype (*n* = 3, mean 65.51%). Within effector CD4+ doublet T cells, a mean of 9.053 % showed expression of both Granzyme B and perforin corresponding with CD4+ CTL (*n* = 3) (Figures 2E–G). Regarding CD57 and CD16 markers, a mean of 18.62% of effector CD4+ doublet T cells were positive for both markers, compared to 65.84% of effector CD8+ doublet T cells (*n* = 3) (Figure 2H). No significant differences were observed between the proportions of naïve, central memory or effector memory subtypes between both groups (*n* = 6).

**Figure 3:**
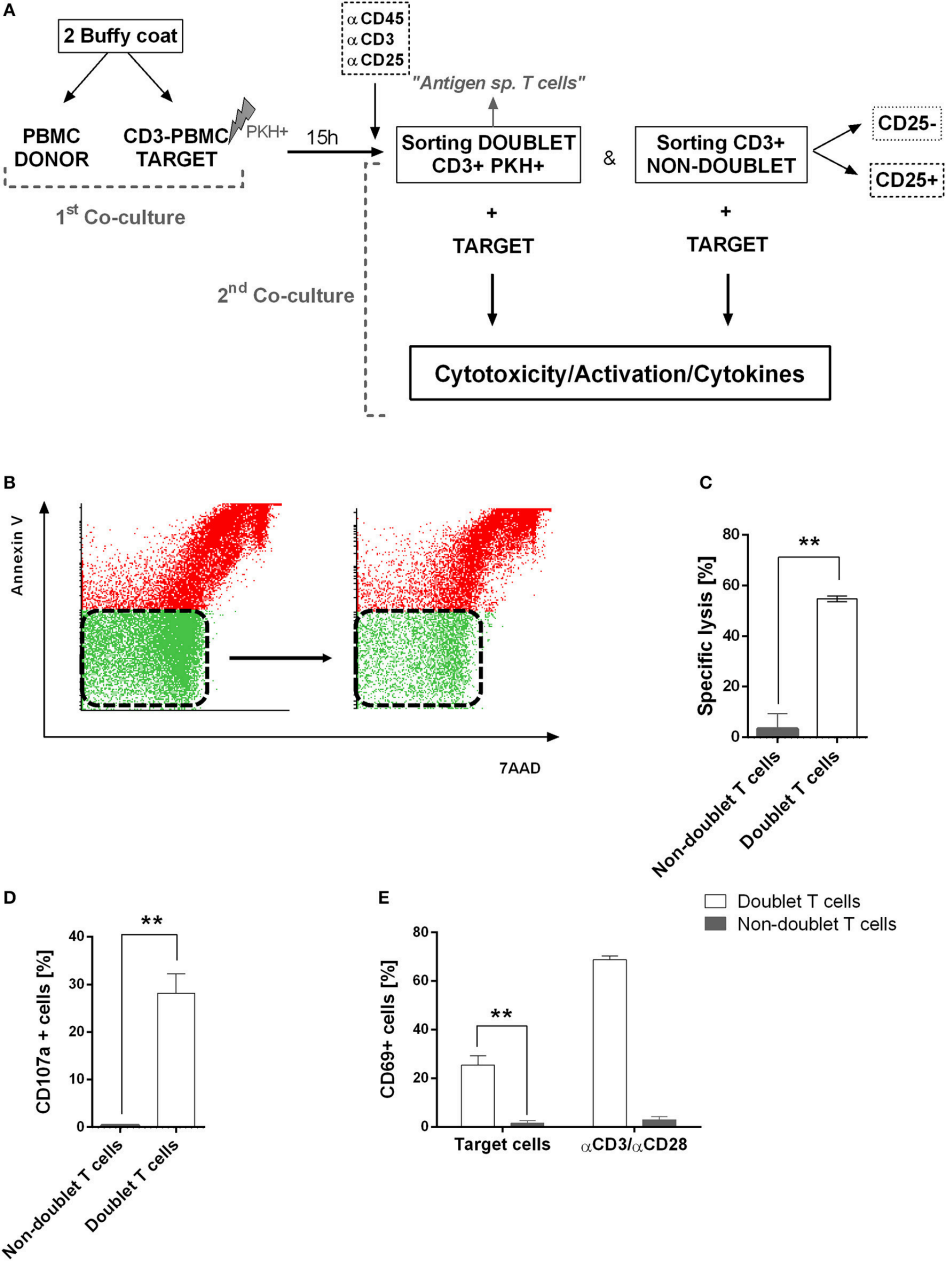
Functional assays of doublet-forming T cells**. (A)** Schedule of the procedure: PBMC from donor were co-cultured with target PBMC depleted of CD3 cells, irradiated and stained with PKH-67 (first co-culture). After 15 h, cells were stained with CD3, CD45, and CD25 monoclonal antibodies for cell sorting. Doublet positive T cells (CD3+PKH+) and non-doublet T cells (CD3+PKH–) were sorted and rested for 20 h. Target cells were thawed and stained with PKH-67 and secondary co-cultures between doublet-forming T cells or non-doublet T cells with target cells were performed (second co-culture). After 7 h, cells were collected and stained with 7AAD and Annexin V. **(B)** Dot plots show 7AAD and Annexin V staining of target cells alone (left) or co-cultured with doublet T cells (right) in one case as an example of analyses performed. **(C)** The bar diagram shows the specific lysis of doublet-forming T cells vs. non-doublet T cells. The specific lysis was calculated following the next formula: [(target viability alone–target viability with doublet or non-doublet T cells)/ target viability alone] × 100. Data show mean values ± *SD* of six independent experiments. **(D)** The bar diagram shows the expression of CD107a cytotoxic marker of doublet-forming T cells vs. non-doublet T cells. Data show mean values ± *SD* of three independent experiments. **(E)** The bar diagram shows the percentage of T cell activation marker CD69 on secondary co-cultures after 24 h of incubation as determined by flow cytometry. Data show mean values ± *SD* of three independent experiments. *P*-values between the indicated groups were calculated using paired Student *t*-tests. **p < 0.01.

**Figure 4:**
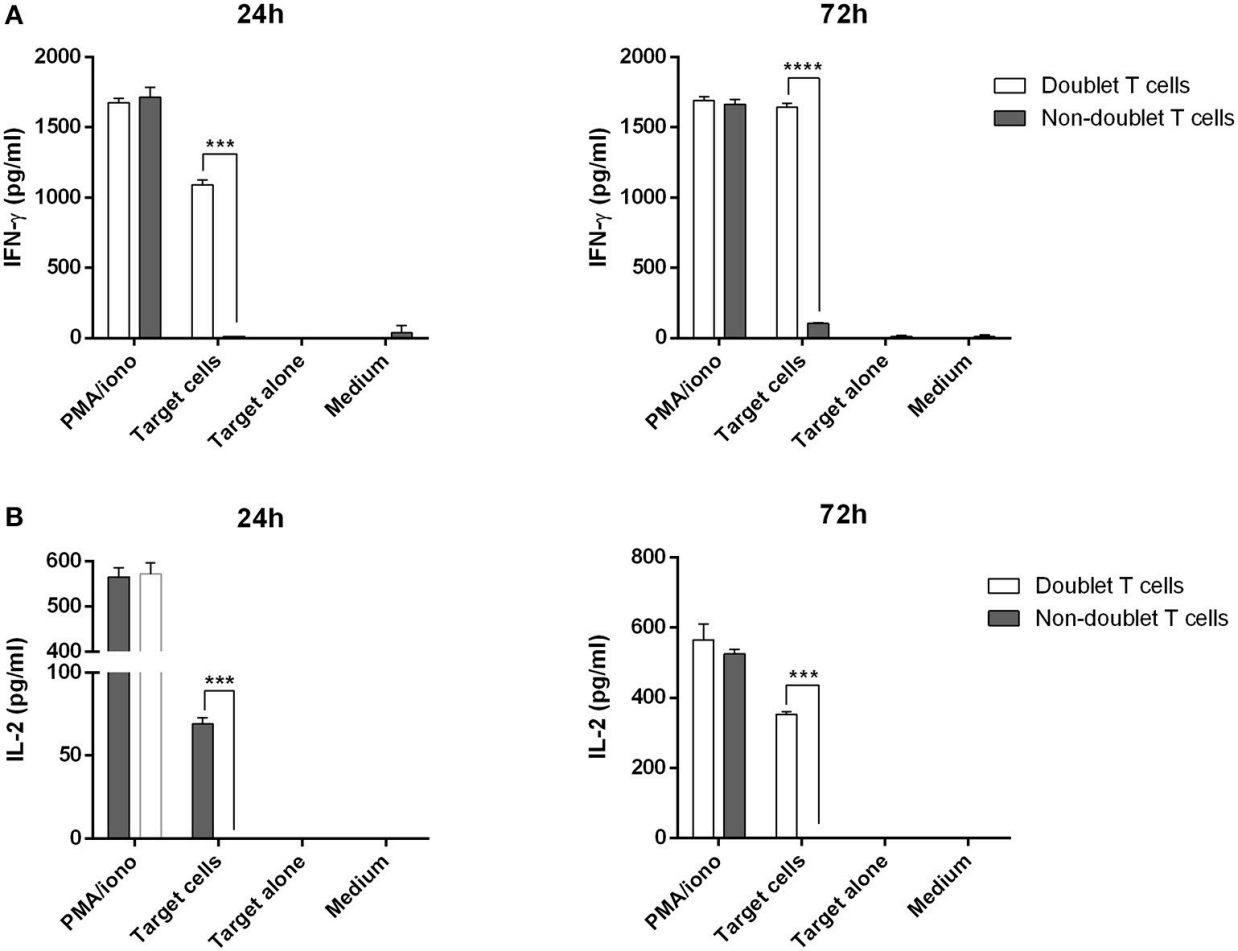
Cytokine production of doublet-forming T cells. Doublet T cells and non-doublet T cells were co-cultured with target cells for 24 and 72 h. **(A)** IFN-γ and **(B)** IL-2 levels in supernatants were measured by ELISA (stimulation performed in duplicates). Data show mean values ± *SD* of one representative experiment. *P*-values between the indicated groups were calculated using paired Student *t*- tests. ***p < 0.001 and ****p < 0.0001.

Next, we were interested in analyzing the cytotoxic activity of doublet-forming T cells (CD3+PKH+) as compared to non doublet-forming T cells (CD3+PKH–CD25–) in secondary co-cultures (Figure 3A). Thus, the sorted populations were again co-cultured with target cells. Therefore, doublet T cells (CD3+PKH+) as well as non-doublet T cells (CD3+PKH–CD25–) were washed and rested for at least 20 h after sorting. During this time, doublet-forming T cells became single T cells due to the elimination of the target cells. CD3-depleted target PBMC were thawed and stained with PKH-67 (CD3–PKH+PBMC). Secondary co-cultures between doublet-forming T cells from donor and target CD3–PKH+PBMC were performed. Of note, target cells in secondary co-cultures were not irradiated in order to analyze the cytotoxic effect of donor's T cells. Live target cells were determined by flow cytometry as 7AAD and Annexin V negative population (Figure 3B). The cytolytic activity was evaluated comparing the viability of target cells cultured alone or with doublet-forming T cells or non-doublet T cells. As shown in Figure 3C, a significant increase of the specific lysis of target cells was obtained when doublet T cells were co-cultured compared to non-doublet T cells (*n* = 6, *p* = 0.0029). Accordingly, the expression of CD107a cytotoxic marker was significantly higher in doublet T cells compared to non-doublet T cells (Figure 3D). Further, we performed secondary co-cultures to analyze the CD69 activation marker after 24 h of co-culture (Figure 3E). A high percentage of CD69+ cells was observed in co-cultures with doublet-forming T cells against target cells as compared to non-doublet T cells (*n* = 3, *p* = 0.0053). When the activation was achieved using anti-CD3/anti-CD28 antibodies, the percentage of CD69+ cells was even higher indicating that the activation against target cells was specific. Finally, analysis of supernatants obtained after a 24 and 72 h of co-culture of doublet T cells and non-doublet T cells with target cells revealed specific secretion of IFNγ and IL-2 (*n* = 3, *p* = 0.0001 at 24 h; *p* = 0.0005 at 24 h, respectively) (Figures 4A,B). Collectively, these data show that doublet-forming T cells isolated by FACS-based cell sorting are specific and effective against target cells.

### A subset of non-doublet T cells has immunosuppressive function

Non-doublet T cells (CD3+PKH–) were sorted based on their CD25 expression. Regarding the CD25+ T cells (CD3+PKH–CD25+), they showed regulatory phenotype expressing CD4, FoxP3, and CD25 markers, but not CD127, thus suggesting that they are regulatory T cells (Figure S4A) (>95%, *n* = 6).

Next, we investigated whether these non-doublet regulatory T cells showed suppressive capacity using functional assays. For this purpose, freshly isolated PKH-67 stained T cells (effector T cells) were stimulated with anti-CD3 and anti-CD28 antibodies and co-cultured with non-doublet regulatory T cells. As controls, effector T cells were cultured alone, either unstimulated or stimulated with anti-CD3/anti-CD28 (Figure S4B). Furthermore, we were interested in studying the suppressive function of the population CD3+PKH–CD25–. Thus, escalating ratios of both non-doublet T cells and effector T cells were performed in order to evaluate the suppressive capacity of these cells. After 4 days of co-incubation, we analyzed the proliferation of effector T cells (Figure S4C). The number of proliferating cells, as assessed by PKH fluorescence diminution, significantly decreased when co-incubated with non-doublet regulatory T cells (CD3+PKH–CD25+) (*n* = 3, *p* = 0.0369 for ratio 1:2; *p* = 0.0150 for ratio 1:1). Surprisingly, non-doublet CD25– T cells (CD3+PKH–CD25–) also showed suppressive function (*n* = 3, *p* = 0.0058 for ratio 1:2; *p* = 0.0151 for ratio 1:1) although at a lower extent. Accordingly, the CD25 activation marker expression was also significantly decreased when non-doublet regulatory T cells or non-doublet CD25– T cells were co-incubated with effector T cells (Figure S4C). In summary, we show that the non-doublet forming T cell fraction is enriched in regulatory T cells, which is in accordance to previous studies indicating that weak interactions between T cells and target cell favor a tolerogeneic immune response.

### Identification and isolation of tumor-specific T lymphocytes from peripheral blood of AML patients

**Figure 5:**
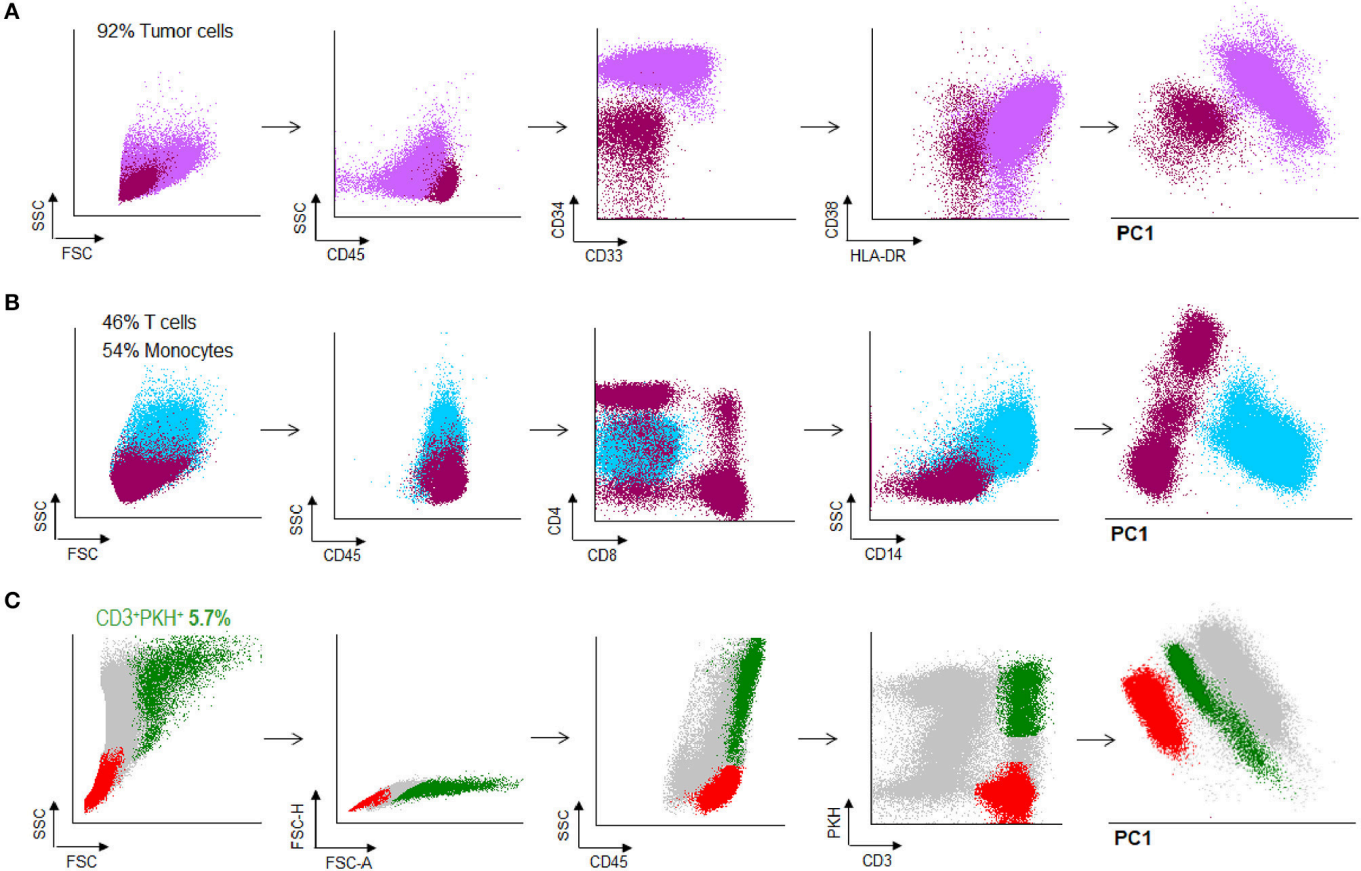
Doublet-forming T cells from peripheral blood of AML patients**. (A)** Blast cells from bone marrow of an AML patient were obtained and frozen. The immunophenotype of AML cells was: CD45low CD34++, CD38+, HLA-DR+ (purple population). **(B)** PBMC were obtained from peripheral blood when the patient was in CR and analyzed by flow cytometry. T cells represented the 46% of the total PMBC. (dark pink population) **(C)** T cells from the patient were co-incubated with AML cells. Doublet-forming T cells were identified based on their FSC/SSC characteristics, doublet zone FSC-A/FSC-H as well as their positivity for CD45 and both PKH and CD3 (CD3+PKH+; green population). Non-doublet T cells were also isolated to use them as control (CD3+PKH–; red population); one representative case is shown.

Bone marrow (BM) samples were obtained from patients with AML. The percentage of tumor cells in the BM samples was >90% in all cases (Figure 5A). After treatment, patients with < 5% blasts in the BM, recovery of neutrophils and platelets, and absence of extramedullary disease were considered in complete remission status (Figure 5B). Under this criterion, PBMC from AML patients in CR were obtained and co-cultured with PKH-67 stained and irradiated autologous tumor cells. After 15 h of co-incubation, cells were stained and harvested for sorting. Doublet-forming T cells from AML patients were identified in a range of 2–6% (Figure 5C) (mean = 3.83%, *n* = 5).

### Doublet-forming T cells from AML patients show specific cytotoxic activity against primary AML blast cells and can be identify in the bone marrow

**Figure 6:**
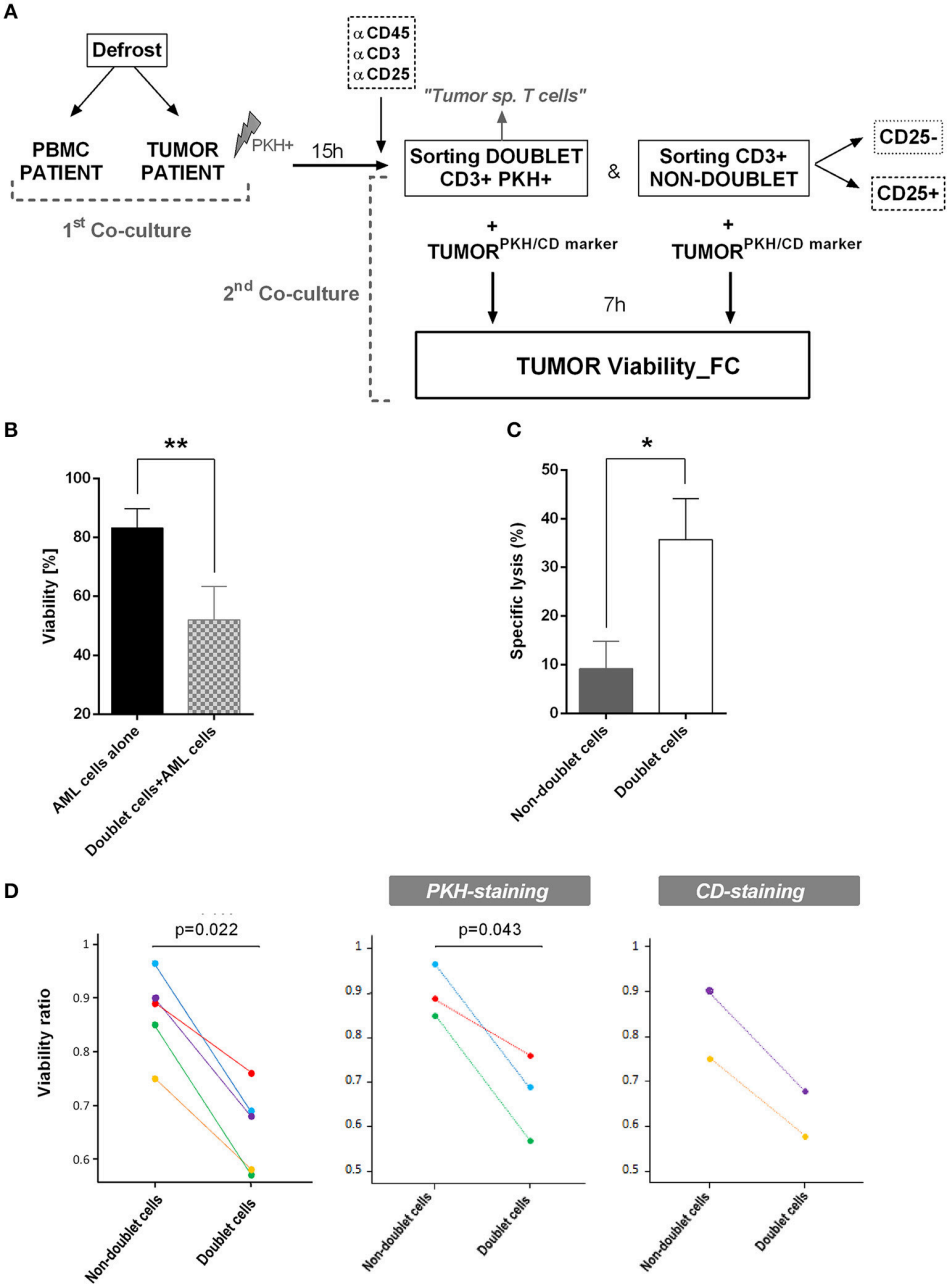
Specific lysis of doublet-forming T cells against AML cells**. (A)** Schedule of the procedure: Tumor cells and PBMC from AML patients were obtained. Tumor cells were irradiated at 25Gy and stained with PKH-67. Co-culture of PBMC and tumor cells from the same patient was performed for 15 h (first co-culture). Then, cells were stained and harvested for cell sorting. The sorted populations (CD3+PKH+ and CD3+PKH–) were rested overnight. Autologous tumor cells were stained with PKH-67 or specific tumor CD markers. Secondary co-cultures between doublet-forming T cells or non-doublet T cells with tumor cells from the same patient were performed (second co-culture). After 7 h, cells were collected and stained with 7AAD and Annexin V for cytometry analysis. **(B)** The bar diagram shows the viability of AML cells alone or co-cultured with doublet-forming T cells. Data show mean values ± *SD* of five independent experiments. **(C)** The bar diagram shows the specific lysis of doublet-forming T cells vs. non-doublet T cells. The specific lysis was calculated following the next formula: [(tumor viability alone–tumor viability with doublet or non-doublet cells)/ tumor viability alone] × 100. Data show mean values ± *SD* of five independent experiments. **(D)** The diagrams show the viability ratio of all cases (PKH-staining + CD-staining, *n* = 5) and the viability ratio of PKH-staining cases (*n* = 3) or CD-staining cases (*n* = 2). The viability ratio was calculated as follows: 1-[(tumor viability alone–tumor viability with doublet or non-doublet cells)/ tumor viability alone]. *P*-values between the indicated groups were calculated using paired Student *t*-tests. *p < 0.05,**p < 0.01.

After identifying doublet T cells from AML patients, we were interested in evaluating the antileukemic effect of sorted doublet-forming T cells. Tumor cells from the same patient (autologous tumor cells) were thawed and stained with PKH-67. Secondary co-cultures of doublet-forming T cells and non-doublet T cells with autologous tumor cells were performed for 7 h (Figure 6A). Of note, tumor cells were not irradiated in order to analyze the cytotoxic effect of doublet-forming T cells from the patient. To determine the cytotoxic activity of doublet-forming T cells, the tumor viability was analyzed by flow cytometry using 7AAD and Annexin V staining. In presence of doublet T cells, the viability of AML cells significantly decrease (Figure 6B) (*p* = 0.0067, *n* = 5). Accordantly, the cytolytic activity was evaluated comparing the viability of tumor cells cultured alone or with doublet-forming T cells or non-doublet T cells from the same patient. As shown in Figure 6C, a significant increase of the specific lysis of AML cells was observed when doublet T cells were co-cultured as compared to non-doublet T cells (*p* = 0.0424, *n* = 5). To further verify the specific cytotoxic activity of doublet-forming T cells, autologous tumor cells in the secondary co-cultures were stained with tumor specific markers based on the immunophenotype of the AML at diagnosis instead of PKH-67. For that, autologous AML cells were thawed and cultured with doublet-forming T cells and non-doublet T cells. After 7 h, cells were stained with tumor specific markers (e.g., CD9, CD34) and tumor viability was analyzed by flow cytometry using 7AAD and Annexin V staining. We finally calculated the viability ratio using both approaches (PKH and CD staining) and a significant difference between doublet-forming T cells and non-doublet T cells was observed in both conditions (Figure 6D).

**Figure 7:**
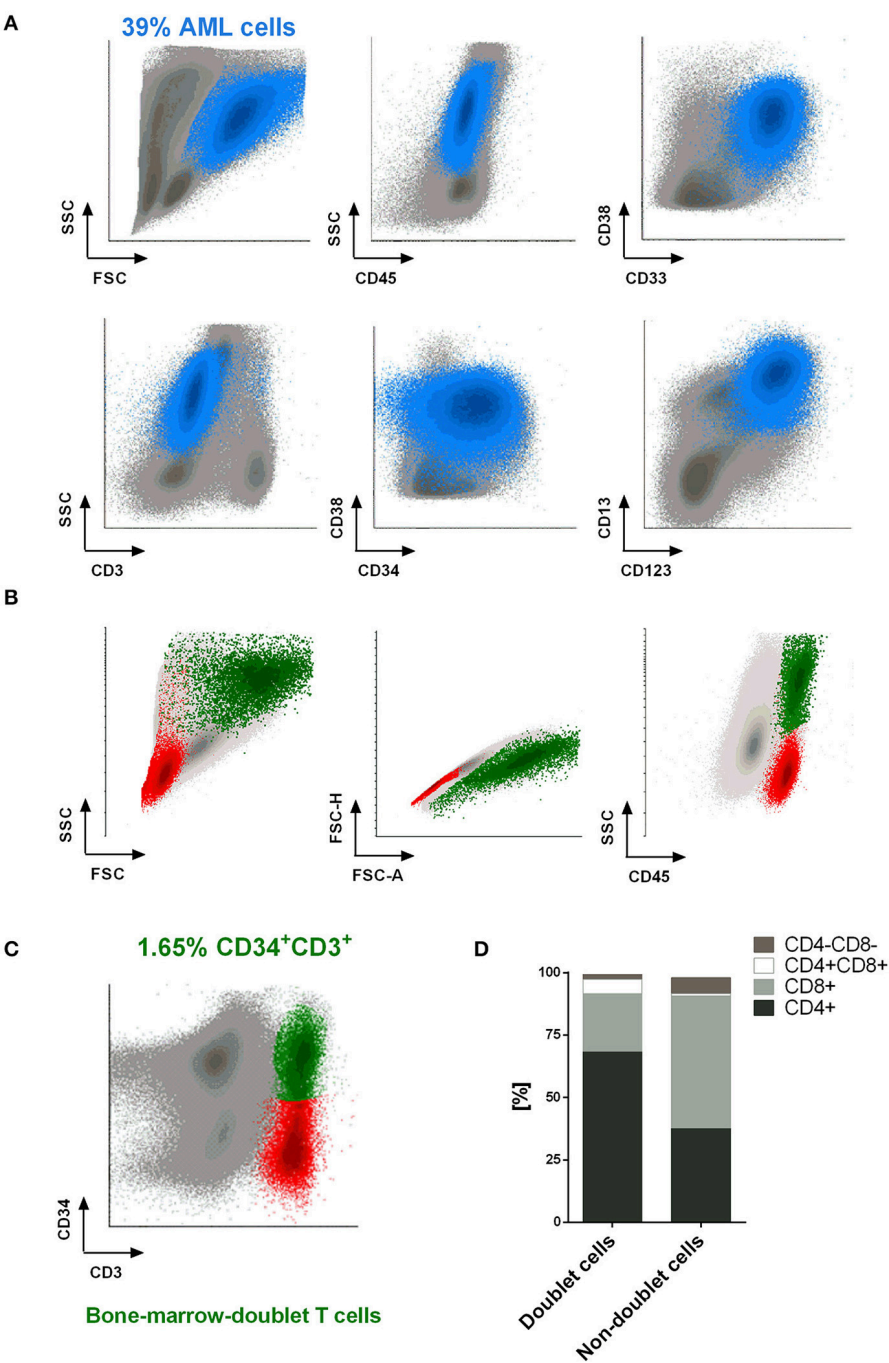
Doublet-forming T cells from bone marrow of AML patients. **(A)** Blast cells from bone marrow of an AML patient are shown. The immunophenotype of AML cells was: CD45low CD34+ CD117+ CD33+ CD38+ CD123+ CD13+/– (blue population). **(B,C)** T cells from bone marrow of an AML patient at diagnose were co-incubated with AML cells. Doublet-forming T cells were identified based on their FSC/SSC, doublet zone FSC-A/FSC-H distribution as well as their positivity for CD45 and both CD3 and CD34 (CD3+CD34+; green population). Non-doublet T cells were also identify as control (CD3+CD34–; red population); one representative case is shown. **(D)** Percentage of CD4+, CD8+, CD4+/CD8+, and CD4–/CD8– cells in bone marrow-doublet T cells compared to bone marrow-non-doublet T cells. Data show mean values of three independent experiments.

This encouraged us to examine whether we were able to identify doublet-forming T cells from bone marrow of AML patients at diagnosis. Analyses of bone marrow by flow cytometry reveled a small percentage of CD3+CD34+ population corresponding with bone marrow-doublet-forming T cells (*n* = 3, mean = 2.9%) (Figures 7A–C). Interestingly, bone marrow-doublet-forming T cells show a higher percentage of CD4+ T cells, whereas bone marrow-non-doublet T cells show a higher percentage of CD8+ T cells (Figure 7D).

In summary, our data demonstrate that when T cells from AML patients are co-cultured with tumor cells, a doublet T cell population appears. This population consists of T cells capable to bind tumor cells. We have shown that CTLs can then be selected and isolated through FACS-based cell sorting. The CTLs from AML patients obtained with this technique display cytolytic activity against AML blast cells suggesting the potential clinical use of these CTLs.

## Discussion

Harnessing the immune system to recognize and destroy tumor cells has been the central goal of anti-cancer immunotherapy. Currently, there is an increasing interest to optimize anti-tumor technologies in order to develop clinically feasible therapeutic approaches. One of the main treatment modalities in cancer immunotherapy is based on adoptive transfer of tumor-specific cytotoxic T cells into cancer patients with the goal of recognizing, targeting, and destroying tumor cells (2, 34). The conventional methods to identify and isolate tumor-specific CTLs are based on cytokine production assays and soluble pMHC multimers (16, 21, 35). The first one is based on selecting those CTLs that highly release INF-γ upon exposure to a specific antigen. However, cytokine-producing T cells are not necessarily cytotoxic, so that they would not present the capacity to destroy target cells. Furthermore, it must be considered the “bystander effect.” This effect is an antigen non-specific stimulation due to some T cells which could release INF-γ not because they are tumor-reactive cells, but because they are stimulated through cytokines released from bystander lymphocytes (18, 19, 36). Prior studies showed that IFN-γ secretion and cytotoxic ability are regulated independently. Thus, the secretion of IFN-γ without killing by some CD8+ T cells was confirmed by combining the Lysispot with an IFNγ Elispot in a two-color assay (19). On the other hand, the use of soluble pMHC multimers consist of multiple pMHC complexes that have been chemically linked together and conjugated to a detectable marker (22). Although, this technology has been successfully used (20, 21), there are several obstacles that need to be solved. For example, the binding affinity threshold for pMHC class I (pMHC-I) tetramers is significantly higher than that required for T cell activation. As a result, pMHC-I tetramers cannot be used to detect all antigen-specific CD8+ T cells (22, 23). Moreover, pMHC multimers can fail for isolation of antigen-specific CD4+ T cells due to the lower affinity of pMHC-II tetramers (22, 24, 37). In this study, we show the feasibility to isolate tumor-specific CTLs from acute myeloid leukemia (AML) patients. We have already published that pHLA-TCR interactions that involve reactive peptides are more stable and strong compared to non-reactive interactions. Thus, we note a significant loss of salt bridges in the non-reactive ternary complexes relative to the reactive complex. This may explain why the interaction between HLA and TCR is weaker in non-reactive complexes than in the reactive ones (31). In this regard, it has been widely described how the dynamics of the interaction between T cells and antigen presenting cells (APC) dictate the quality of the immune responses. While stable joints between target cells and T lymphocytes lead to the induction of T cell activation and immune response, brief contacts contribute to the induction of immune-tolerance (29).

Taking advantage of the strong interaction between target cell and activated T-cells, we sought to isolate functional tumor-specific CTLs through FACS-based cell sorting. First, we began by performing co-cultures using PBMC from two different healthy donors to set up the best conditions to identify and select those T-cells which recognize and bind the target cell (doublet-forming T-cells). After stablishing the cell culture and sorting strategies, we translate these conditions to the autologous setting in patients with AML. Although cell-cell interaction could be stronger in the allogeneic than in the autologous setting, we could identify and isolate doublet-forming T-cells in AML patients through FACS-based cell sorting. In fact, we observed that when T cells from AML patients are co-cultured with autologous tumor cells, doublet population can be identified. This population consists of T-cells bound with strong interactions to tumor cells. Thus, following the sorting strategy previously described, we were able to isolate tumor-specific CTLs from AML patients. These AML-specific CTLs show cytolytic activity against primary blast cells suggesting their potential use in the clinical setting. In this regard, different applications have been recently developed in order to perform sorting in a fully enclosed and sterile cartridge system without sheath fluid for medical applications. Using a similar approach, the current proposal to identify doublet forming T-cells could be transferred to the clinic. Interestingly, not only CD8+ tumor-reactive T cells are isolated with this approach, but also effector CD4+ CTLs. Although CD4+ T cells are classically viewed as helper cells facilitating CD8+ T cell function, it is now clear that both cell subsets can exert cytotoxicity against tumor targets (3, 38). Moreover, we have demonstrated that by sorting doublet-forming T cells, we are selecting only cytotoxic T cells and depleting regulatory T cells with immune suppressive function from the pool of patient's T cells.

Therefore, “Doublet Technology” is a fast, cost-effective approach to identify autologous tumor-reactive T cells from acute myeloid leukemia patients. The principal advantage of this strategy is that there is no need for *a priori* knowledge of exact tumor antigen, emphasizing the broadly applicability of this technology. Recently, the existence of TIL have been described in patients with hematologic malignancies, more specifically, marrow-infiltrating lymphocytes (MIL) have been identified in multiple myeloma patients (38, 39). Our data demonstrate for the first time that using this approach, we are able to identify tumor-specific CTLs from peripheral blood and bone marrow of AML patients. Interestingly, bone marrow-doublet-forming T cells show a higher percentage of CD4+ T cells as compared to peripheral blood doublet-forming T-cells. In line with our data, a recent report has shown that TIL fragments derived from small pieces of the patient's tumor contain more CD4+ T cells than CD8+ T cells (19/24 TIL fragments). In fact, after expansion, the infused cells were predominantly CD4+ T cells (62.5%) and they mediated the complete durable regression of metastatic breast cancer, which is now ongoing for >22 months (40). In this sense, further studies are needed to evaluate the cytotoxic activity of bone marrow doublet-forming T-cells and compare it to peripheral blood doublet-forming T-cells. Additionally, the identification of tumor antigens is needed for the development of new therapeutic strategies against cancer. Thus, we could take advantage of the “Doublet technology” to sequence and clone the region CDR3 of the tumor-reactive CTLs isolated and to identify the antigens or groups of antigens against which the cytotoxic antitumor response is generated. Moreover, TCR sequence of these natural tumor-reactive CTLs can be used to redirect lymphocyte specificity to cancer antigens by genetic engineering.

In conclusion, we present “Doublet Technology” as a novel approach which allows to identify and isolate functional tumor-specific T cells from patients diagnosed with acute myeloid leukemia.

## Author contributions

EG-G designed and performed experiments, analyzed data and wrote the manuscript. LS-A, ED, and TR performed experiments and analyzed data. JB-G, JG-C, and TC-V provided biologic material. JP-S designed experiments and project.

### Conflict of interest statement

EG-G, JP-S, and LS-A are co-inventors on a patent application related to this work that has been filed by the Virgen del Rocio University Hospital. The remaining authors declare that the research was conducted in the absence of any commercial or financial relationships that could be construed as a potential conflict of interest.

## Supplementary material

Figure S1 Cell culture conditions and sorting of doublet-forming T cells and non-doublet T cells. **(A)** The boxplots show the percentage of donor T cell activation markers (CD25 and CD69) after 48 h of co-culture as determined by flow cytometry. Shown are mean ± *SD* of four independent experiments. *P*-values between the indicated groups were calculated using paired Student *t*-tests. ^**^*p* < 0.01. Donor T cells were gated based on the expression of CD45+, CD3+, and CD19–. **(B)** Co-cultures of donor's PBMC and target CD3–PKH+PBMC were performed at a ratio of 3:1 for 15 h. After incubation, cells were stained, harvested and sorted. The population with viable, higher FSC/SSC, doublet zone FSC-A/FSC-H distribution was selected. Double positive cells (CD3+PKH+) and non-doublet T cells (CD3+PKH–) were gated. Within the non-doublet T cells, two different populations were sorted using the antibody CD25 (CD3+PKH–CD25+ and CD3+PKH–CD25–). The nozzle size used to sorter the doublet cells was 85 μm.

Figure S2 Purity of doublet-forming T cells and non-doublet T cells. **(A)** The dot plot shows the doublet-forming T cells (CD3+PKH+) and non-doublet T cells (CD3+PKH–) before the sorting procedure. **(B)** Dot plots show the isolated cells (CD3+PKH+ and CD3+PKH–) after the sorting procedure. One representative case is shown.

Figure S3 Ratio CD4/CD8, percentage of effector T cells and cytotoxic markers in doublet-forming T cells. **(A)** The diagram shows the percentage of CD4+ and CD8+ T cells in doublet population vs. non-doublet population. The mean percentage of CD4+ cells in doublet and non-doublet population was 25.73 vs. 65.42%, respectively. The mean percentage of CD8+ cells in doublet and non-doublet population was 50.86 vs. 23.42%, respectively. **(B,C)** The diagrams show the percentage of effector CD4+ and CD8+ cells. The mean percentage of effector CD4+ cells in doublet and non-doublet population was 5.57 vs. 1.47%, respectively. Regarding effector CD8+ cells, the mean percentage comparing doublet and non-doublet population was 19.57 vs. 12.43%, respectively. Depicted are the mean ± *SD* of six independent experiments. *P*-values between the indicated groups were calculated using paired Student *t*-tests. ^*^*p* < 0.05 and ^***^*p* < 0.001. **(D)** Dot plots show the expression of Granzyme B (upper panel) and perforin (bottom panel) on effector CD4+ and CD8+ doublet T cells from one case as an example of analyses performed (n = 3 experiments). **(E)** Dot plots show the expression of CD57 (upper panel) and CD16 (bottom panel) on effector CD4+ and CD8+ doublet T cells from one case as an example of analyses performed (*n* = 3 experiments).

Figure S4 Immunophenotype and suppression assays of non-doublet T cells. **(A)** The regulatory phenotype was evaluated in non-doublet T cells that express CD25. Dot plots show the expression of CD4, CD25, FoxP3, and CD127 on non-doublet T cells CD25+ (CD3+PKH–CD25+). Data show one representative experiment of six independent experiments. **(B)** Proliferation, monitored by PKH-67 dilution of control or αCD3/αCD28 stimulated responder T cells, co-incubated or not with non-doublet T cells; one representative case is shown. **(C)** The percentage of proliferation (upper bar diagram) and CD25 expression (bottom bar diagram) of effector T cells is shown. Proliferation was assessed by PKH fluorescence using ModFit software. PKH and CD25 expression were analyzed by flow cytometry. Depicted are the mean ± *SD* of three independent experiments. *P*-values between the indicated groups were calculated using paired Student *t*-tests. ^*^*p* < 0.05 and ^**^*p* < 0.01.
